# Development of Peritoneal Carcinoma in women diagnosed with Serous Tubal Intraepithelial Carcinoma (STIC) following Risk-Reducing Salpingo-Oophorectomy (RRSO)

**DOI:** 10.1186/s13048-019-0525-1

**Published:** 2019-05-25

**Authors:** P. I. Stanciu, T. E. J. Ind, D. P. J. Barton, J. B. Butler, K. M. Vroobel, A. D. Attygalle, M. A. E. Nobbenhuis

**Affiliations:** 1 Gynaecological Oncology Department, The Royal Marsden Hospital, The Royal Marsden NHS Foundation Trust, Fulham Road, London, SW3 6JJ UK; 2 Pathology Department, The Royal Marsden Hospital, London, UK

**Keywords:** BRCA, Serous tubal intraepithelial carcinoma, Risk-reducing Salpingo-oophorectomy, Peritoneal carcinoma

## Abstract

**Introduction:**

The management of Serous Tubal Intraepithelial Carcinoma (STIC) found at the time of Risk-Reducing Salpingo-Oophorectomy (RRSO) remains unclear. We set out to analyse the incidence of peritoneal carcinomas developed after prophylactic surgery and to formulate further guidance for these patients.

**Methods:**

This is a retrospective study of 300 consecutive RRSO performed at the Royal Marsden Hospital between January 2008 and January 2017.

**Results:**

The median age at RRSO was 47.8 years (range 34 to 60 years) and median BMI was 26.2 kg/m^2^ (range 16 to 51 kg/m^2^). A total of 273 patients (91%) were tested for BRCA mutations. Of these, 124 (45.4%) had a BRCA 1 mutation, 118 (43.2%) had a BRCA 2 mutation, 2 (0.7%) had both a BRCA 1 and a BRCA 2 mutation and 29 (10.6%) had no BRCA mutation detected. Isolated STIC lesions were identified in 7 cases (2.3%) and p53 signatures in 75 cases (25%). There were five (1.6%) incidental tubal carcinomas and one (0.3%) ovarian carcinoma at the time of surgery. Two (28.6%) of the 7 patients with STIC identified following RRSO had high grade serous peritoneal carcinoma diagnosed at 53 and 75 months. One (0.3%) patient from the other 287 patients from our series with no STIC diagnosis or incidental carcinomas at RRSO developed high grade serous carcinoma of peritoneal origin after 92 months.

**Conclusion:**

This study demonstrates that when a STIC lesion is identified following RRSO there is a significantly higher risk of a subsequent peritoneal cancer. Although there is no published consensus in literature, we recommend that consideration should be given for long term follow-up if a STIC lesion is identified at RRSO.

## Introduction

In the last few decades a growing number of women are being diagnosed with genetic mutations that increase their lifetime risk of developing ovarian cancer. The main ovarian cancer subtype is high grade serous carcinoma (HGSC) and almost 20% of these are associated with germline mutations of BRCA 1 and 2, but more than 40% of the patients do not have a family history of cancer and are not routinely tested [1]. Overall, women with BRCA 1 and 2 germline mutations have a higher risk of developing ovarian cancer (44 and 17%, respectively) compared with the 1 to 5% baseline risk of the general population [2]. With high grade serous being a major histological subtype, this population has also an increased risk for serous uterine and peritoneal cancers [3].

HGSC of the ovary usually present as advanced stage, progress rapidly and have a poor prognosis. There is increased evidence that the distal part of the fallopian tube is the site of origin of most ovarian and peritoneal HGSC and Serous Tubal Intraepithelial Carcinoma (STIC) represents the precursor lesion, although it is believed that STIC itself is likely to have metastatic potential [4, 5]. A large spectrum of fallopian tube lesions have been described ranging from p53 signatures with normal-appearing tubal epithelium, no atypia and low proliferation index based on Ki-67 immunostaining but with mutant p53 expression pattern [6] to serous tubal intraepithelial lesions (STIL) with cytological atypia but falling short of STIC [7]. p53 immunohistochemistry is one of the most efficient markers for STICs and a predictor for TP53 mutation. More than 90% of STICs have p53 signatures and share identical TP53 mutations with invasive ovarian or peritoneal cancers suggesting that STICs are the site of origin for high grade serous carcinomas [8]. Germline TP53 mutations are considered to have a significant role in the oncogenesis of several human cancers [9] and are found in more than 95% of ovarian high grade serous carcinomas [10]. These mutations represent the initial event in the development of high grade serous carcinomas and are documented in the majority of STICs [11, 12].

For women at increased risk, management options include ovarian screening and prophylactic surgery. Currently there is no widely accepted screening strategy for ovarian cancer, [13] but several studies have proven the effectiveness of Risk-Reducing Salpingo-Oophorectomy (RRSO), showing a reduction of approximately 80% in the risk for developing ovarian or fallopian tube cancer after prophylactic surgery [14–16]. Apart from reducing ovarian cancer risk, RRSO has been proven to reduce overall mortality up to 60% and is strongly recommended for prevention of ovarian cancers in BRCA 1/2 mutation carriers [14, 17].

As removal of the ovaries in pre-menopausal patients increases the risk for osteoporosis, coronary disease, stroke and total mortality, RRSO is not recommended in the general population [18]. For BRCA1 carriers RRSO is recommended before the age of 40 and for BRCA 2 carriers RRSO is recommended before the age of 45 or in any case when their family is complete [19]. Even with this strategy, the risk for development of subsequent peritoneal carcinomas has been reported to be 1 to 4.3% [14, 20].

The incidence of occult high grade serous carcinomas in the distal fallopian tube at the time of RRSO varies from 5 to 15% [21, 22] and the incidence of STIC varies from 0.6 to 7% in women with BRCA1 and BRCA2 mutations [23].

The aim of this study is to review the histological findings of 300 consecutive RRSOs, to analyse the association of STIC with subsequent diagnosed peritoneal carcinomas and to discuss the possible change in the management and follow up of these patients.

## Materials and methods

This is a retrospective study of 300 consecutive RRSOs performed at the Royal Marsden Hospital (RMH) in London, between January 2008 and January 2017.

### Patient selection

Eligible patients were referred to the Department of Gynaecological Oncology Surgery by the Department of Genetics. Inclusion criteria were as follow: patients undergoing RRSO at RMH between 01/01/2008–01/01/2017; strong family history of breast or ovarian cancer and/or BRCA carriers; surgery undertaken by RMH Gynaecological Oncology Surgeon; pathology and cytology data available on Electronic Patient Record (EPR) database. Patients with suspected or known gynaecologic cancer were excluded from the study.

### Technique of RRSO

All RRSO procedures were performed laparoscopically. Peritoneal washings and endometrial sampling were performed as standard when technically possible. Histopathological assessment was undertaken which included processing and entirely submitting the fallopian tubes according to the SEE-FIM protocol [24]. Immunohistochemistry for p53 and MIB1 were also performed on the fimbrial ends of the fallopian tubes to identify any p53 signatures or other small lesions not identified on the initial H + E sections. Where submitted, histological examination of the endometrium samples was carried out to exclude concurrent endometrial pathology. Cytology of the peritoneal fluid was performed to identify the presence of atypical or malignant cells.

### Data selection

Demographic data, family history, genetic testing, preoperative CA125 and pelvic scans were extracted from EPR.

### Follow-up

Apart from patients with incidental findings of occult gynaecological cancers at RRSO, all other patients (including patients with histological confirmation of STIC) were discharged from gynaecological care considering that during the period these cases were operated on there was no consensus on clinical significance and appropriate management of isolated STICs. As the patients remained under Breast Team follow up at RMH, we were able to investigate any new cancer developed by reviewing their medical records on EPR. All data are presented as descriptive statistics (number of observations and percentages). Fisher exact test was used for statistical analysis. The results were considered significant at *p* < 0.05.

## Results

The median age of women at the time of RRSO was 47.8 years (range 34 to 60 years) and median BMI was 26.2 kg/m^2^ (range 16 to 51 kg/m2) (Table 1). Out of 91% of the patients tested for BRCA mutations (273 women), 45.4% (124 women) had a BRCA 1 mutation and 43.2% (118 women) had a BRCA 2 mutation. Two women (0.7%) had both a BRCA 1 and a BRCA 2 mutation and 29 women (10.6%) had no mutation detected. Twenty seven patients (9%) from our series declined testing for BRCA mutations and were not tested. Preoperative CA125 was recorded and retrieved from EPR for 291 cases (97%) and it was normal (0–35 u/ml) in 274 cases (94.2%). Out of 17 cases (5.8%) with abnormal (> 35 u/ml) preoperative CA 125, at the time of RRSO we diagnosed one occult ovarian high grade serous carcinoma (CA 125 of 85) and two tubal high grade serous carcinomas (CA 125 of 100 and 36, respectively). No cancer was diagnosed at the time of RRSO in the other 14 cases with elevated CA 125 (median CA 125 was 54, range 36 to 159).

**Table 1:** Demographic characteristics

Characteristics	Number (%)
Median age (years)	47.8
Range	34–60
Median BMI (kg/m^2^)	26.2
Range	16–51
BRCA mutation testing	273 (91%)
Not done/declined	27 (9%)
BRCA1	124 (45.4%)
BRCA2	118 (43.2%)
BRCA 1 and 2	2 (0.7%)
BRCA negative	29 (10.6%)
Preoperative CA125 testing	291 (97%)
Not recorded	9 (3%)
Normal (0–35 u/ml)	274 (94.2%)
Abnormal (> 35 u/ml)	17 (5.8%)
Preoperative pelvic imaging	300 (100%)
Normal findings	296 (98.7%)
Suspicious findings	4 (1.3%)

All patients underwent pelvic ultrasound imaging prior to surgery, with additional CT and MRI when indicated. Suspicious features were detected in 4 cases (1.3%). The first case was an asymptomatic 62 years old female, BRCA 1 carrier, with a large echogenic cyst measuring 9 × 6.5 cm on the left ovary, containing debris suggesting previous haemorrhage. There was no abnormal vascularity seen on colour Doppler and in view of the elevated CA 125 of 100 it was labelled as suspicious and excision was advised. Intraoperative findings were not suggestive of malignancy and a BSO was performed with the specimen extracted in a bag without spillage. The final histology showed high grade serous carcinoma of the left tube (pT1a) with negative cytology and no p53 signatures. In the second case (42 years old female, BRCA 2 carrier), pelvic ultrasound demonstrated two cystic structures within the right ovary and minimal free fluid in the pouch of Douglas. CA 125 was 85 and intraoperatively, suspicious deposits seen in the pouch of Douglas were biopsied and sent for histology along with the BSO specimen. The final histology revealed high grade serous carcinoma of the ovary (with mutant p53 staining pattern) with peritoneal deposits and cytology positive. Bilateral coincidental benign Brenner tumours were seen. Following staging surgery the final FIGO stage was 3A2 due to microscopic high grade serous carcinoma disseminated within the omentum. The third patient was a 53 years old woman who declined BRCA testing, but with a strong family history of ovarian and breast cancer (first and second degree relatives) and a CA 125 of 36. Following an abnormal pelvic ultrasound, she underwent a MRI which demonstrated a small 2.1 × 3 cm right ovarian cyst with enhancing papillary soft tissue suspicious for malignancy. Suspicious peritoneal deposits were biopsied intraoperatively and final histology report confirmed high grade serous carcinoma of both fallopian tubes with a background of serous tubal intraepithelial carcinoma of the left tube (with mutant, over-expressed p53 staining) and infiltration of both ovaries. Peritoneal cytology was positive for malignant cells. Bladder and pelvic peritoneal biopsies showed high grade serous carcinoma and tumour was initially staged as 2B, but upstaged after final surgery to 3A1 due to left external iliac lymph node involvement. For the fourth woman, abnormal pelvic ultrasound demonstrated a bulky left ovarian thin walled cyst, but the final pathology returned benign findings.

In total 6 occult cancers were detected in our series at the time of surgery (2%): one ovarian and five tubal carcinomas, all of high grade serous histological subtype. Peritoneal cytology was performed in all cases and in three cases (1%) malignant cells were diagnosed. Two of them were detailed above (one stage 3A2 high grade ovarian carcinoma and one stage 3A1 fallopian tube carcinoma) and the third case was a 61 years old female, BRCA 2 carrier, with normal preoperative pelvic ultrasound and no suspicious findings at the time of RRSO. Histology report revealed high grade serous carcinoma arising in the right fallopian tube associated with STIC and also involving the left tube and surface of both ovaries, FIGO stage 2A (Table 2).

**Table 2:** Characteristics of incidental carcinomas detected at RRSO

Age	Histology	Cytology	Stage^a^	Preoperative scan	Preoperative CA125 (u/ml)	Concurrent p53 signature	BRCA
62	Tubal HGS Carcinoma	Negative	1A	suspicious ovarian cyst	100	No	1
42	Ovarian HGS Carcinoma	Positive	3A2	right ovarian complex cyst	85	Yes	2
53	Tubal HGS Carcinoma	Negative	1A	normal scan	20	No	2
61	Tubal HGS Carcinoma	Positive	2A	normal scan	24	Yes	2
53	Tubal HGS Carcinoma	Positive	3A1	suspicious ovarian cyst	36	Yes	-^b^
66	Tubal HGS Carcinoma	Negative	1A	normal scan	9	Yes	1

^a^FIGO classification 2014 version

^b^Patient declined testing

Isolated STICs were diagnosed in 7 cases, representing 2.3% of all patients included and in 75 cases (25%) p53 signatures were found. All patients diagnosed with STIC had p53 signatures. Isolated STIL lesions were diagnosed in 2 cases (0.7%), both of which had a normal CA125, one had p53 signature and the other one did not. Endometrial biopsies were performed in 257 cases (85.7%) and in all showed no evidence of carcinoma. In 43 cases (14.3%) endometrial sampling was not possible due to cervical stenosis. None of these cases had suspicious endometrium at scanning, nor subsequently developed uterine malignancies. Endometriosis and benign ovarian cysts were found in 10 (3.3%), respective, 54 (18%) cases. Pathological findings are detailed in Table 3.

**Table 3:** Pathological findings at RRSO

Findings	Number (%)
Peritoneal cytology	300 (100%)
Positive	3 (1%)
Negative	297 (99%)
Endometrial biopsy	257 (85.7%)
Not done	43 (14.3%)
Positive for malignancy	0 (0%)
Negative	257 (100%)
STIC^a^	7 (2.3%)
STIL	2 (0.7%)
P53 signatures	75 (25%)
Tubal cancer	5 (1.6%)
Ovarian cancer	1 (0.3%)
Benign ovarian cysts	54 (18%)
Endometriosis	10 (3.3%)

^**a**^Patients diagnosed with STIC are detailed in Table 4

All the procedures were performed laparoscopically and there were four conversions to laparotomy (1.3%). Morbid adhesions and frozen pelvis were responsible to conversion to laparotomy in three cases and external iliac vein injury resulted in conversion of the fourth case.

Of the seven patients diagnosed with isolated STICs at the time of RRSO, one (14.2%) declined testing and six (85.8%) were tested for BRCA mutations: 3 women (42.9%) were positive for BRCA 1 and 3 women (42.9%) were positive for BRCA 2. Preoperative CA 125 was normal in 6 cases (85.8%) and slightly elevated to 38 in one case (14.2%) but no invasive carcinoma was diagnosed. Peritoneal washings were negative for all 7 cases with STIC and all preoperative scans were normal. STIC lesions were unilateral in all cases. p53 signatures were bilateral in 3 cases (42.9%) and unilateral, corresponding to STIC lesions, in 4 cases (57.1%). (Table 4).

**Table 4:** Characteristics of the patients diagnosed with isolated STIC

Patients with STIC	Number (%)
BRCA testing	6 (85.8%)
Not done/declined	1 (14.2%)
BRCA1	3 (42.9%)
BRCA2	3 (42.9%)
Preoperative CA125 testing	7 (100%)
Normal (0–35 u/ml)	6 (85.7%)
Abnormal (> 35 u/ml)	1 (14.3%)
Preoperative pelvic imaging	7 (100%)
Normal findings	7 (100%)
Suspicious findings	0 (0%)
P53 signatures	
Unilateral	4 (57.1%)
Bilateral	3 (42.9%)
Peritoneal cytology	
Positive	0 (0%)
Negative	7 (100%)

By September 2018, three patients from our series (1%) developed HGSC of the peritoneum. Two of these patients that developed further peritoneal cancer had unilateral STIC lesions diagnosed at the time of RRSO, with bilateral p53 signature in one case and unilateral corresponding p53 signature in the other case. CA125 was normal in both cases. The third patient that further developed HGSC of the peritoneum had no STIC nor p53 signature found at the time of RRSO and had a normal CA125. The incidence of subsequent peritoneal carcinoma in patients with isolated STIC was 28.6% (2/7) compared to 0.3% (1/287) in patients without STIC or occult malignancy. *P* = 0.001 (Table 5).

**Table 5:** Characteristics of uterine and peritoneal carcinomas developed after RRSO

Age	Histology	Cytology	Stage^a^	BRCA	STIC	Concurrent p53 signatures	Time of diagnosis (months after RRSO)	CA125(u/ml) (initial/relapsed)
39	Peritoneal HGS Carcinoma	Negative	3C	1	No	No	92	10/52
41	Peritoneal HGS Carcinoma	Negative	2A	1	Yes	Yes	75	31/5418
58	Peritoneal HGS Carcinoma	Negative	4	2	Yes	Yes	53	9/2957

^a^FIGO classification 2014 version

## Discussion

### STIC

The incidence of isolated STIC in our series was 2.3% (7 cases) and is comparable with the incidence published in the literature, which varies from 0.6 to 7% [23]. Six of these patients tested positive for BRCA mutations (3 patients with BRCA1 and 3 patients with BRCA 2) and one declined testing. In the entire studied group, no patient known to be BRCA negative was diagnosed with STIC and we conclude, as previous indicated by Manchanda et al., that the risk of isolated STIC is low for patients without documented BRCA mutations [25].

All seven STICs had coexisting p53 signatures and two of them (28.6%) developed peritoneal high grade serous carcinomas at 53 and 75 months after RRSO. As the clinical significance of isolated STICs remains unclear and there is no recommended treatment, no further surgical staging and/or adjuvant chemotherapy was undertaken in our series after STIC was diagnosed.

On the one hand, a systematic review looking at the outcomes of isolated STICs at RRSO suggested that additional treatment (further staging and/or chemotherapy) is associated with a smaller recurrence risk, but routine staging was not recommended [26]. On the other hand, a recent comprehensive review of literature failed to establish the role of adjuvant therapy [27].

Wethington et al. [23] also looked at the outcome of 12 BRCA carriers diagnosed with isolated STICs and found no difference in recurrence rate after staging or no treatment. Along with other authors [23, 28, 29] they concluded that this subgroup of patients with isolated STIC diagnosed at the time of RRSO should be offered long term follow up that could include CA 125 testing, but did not comment on the extent of surveillance.

### Peritoneal Cancer

In BRCA carriers, peritoneal high grade serous carcinoma usually presents as advanced stage disease many years after RRSO. Iavazzo et al. [30] looking at 1830 RRSO for BRCA carriers, estimated at 1.53% the lifetime risk of developing primary peritoneal cancer for this subset of patients and found a long interval of time ranging from 12 to 84 months from initial prophylactic surgery to presentation of peritoneal cancer. For this reason they suggested long term follow-up for this patients but did not comment about presence or absence of STICs at the time of RRSO.

The presence of STICs and potential peritoneal seeding of premalignant cells during or before salpingo-oophorectomy was regarded by some authors as a possible mechanism that could explain the origin of peritoneal cancer after prophylactic removal of tubes and ovaries [31]. Our data strongly suggest that the presence of STIC at the time of RRSO is a major predisposing risk factor for subsequent development of peritoneal cancer.

The three patients in our series who developed peritoneal cancer had negative peritoneal cytology at the time of RRSO. Two out of these three (66.7%) had a diagnosis of STIC at the time of prophylactic surgery and taking into consideration presentation at 53 and 75 months, long term follow up could be advocated for BRCA carriers with isolated STICs found at the time of RRSO. Interestingly, although initial CA 125 was less than 35 u/ml, at the time of recurrence CA 125 was grossly elevated in both cases (5418 u/ml and 2957 u/ml) suggesting that adding CA 125 at routine surveillance may be valuable in the earlier detection of peritoneal cancer post-RRSO.

The other patient who developed peritoneal cancer had initial negative washings and no STIC identified within the tube and was also a late presentation at 92 months after prophylactic salpingo-oophorectomy.

## Conclusion

In our single centre series, STIC was associated with a significantly higher risk of subsequent peritoneal high grade serous carcinoma, therefore we now recommend all these patients should be considered for long term surveillance for up to 10 years.

## Data Availability

All patients details are available on Electronic Patient Record database of The Royal Marsden Hospital NHS Trust.
